# Nurses’ perceptions of the performance of nurse managers and senior hospital management during the COVID-19 pandemic

**DOI:** 10.1186/s12912-024-02123-4

**Published:** 2024-07-03

**Authors:** Sara Huerta-González, Pedro Ángel Caro-Alonso, Beatriz Rodríguez-Martín, Carlos A. Castillo-Sarmiento, Juan Diego Pedrera-Zamorano, Carlos Chimpén-López, Sergio Rico-Martín, Fidel López-Espuela

**Affiliations:** 1 Faculty of Nursing, Universidad Veracruzana, Tuxpan, Poza Rica, Mexico; 2 Health Service of Castilla-La Mancha, La Algodonera Health Center, Talavera de la Reina, Toledo, Spain; 3 Department of Nursing, Physiotherapy and Occupational Therapy, Faculty of Health Sciences, University of Castilla-La Mancha. Talavera de la Reina, Toledo, Spain; 4 Department of Nursing, Physiotherapy and Occupational Therapy, School of Physiotherapy and Nursing, University of Castilla-La Mancha, Toledo, Spain; 5 Department of Nursing, Faculty of Nursing and Occupational Therapy, University of Extremadura, Cáceres, Extremadura Spain

**Keywords:** COVID-19, Health manager, Nurse, SARS-COV-2, Supervisory nursing, Qualitative research

## Abstract

**Background:**

The first waves of the COVID-19 pandemic had a negative impact on health systems and health professionals, due to the high number of cases and a lack of preparation. The aim of this study was to understand how nurses working in hospital units and in intensive care perceived the performance of nurse managers and senior hospital management during the first two waves of the pandemic.

**Methods:**

The phenomenological approach proposed by Giorgi was used to investigate perceptions of the performance of nurse managers and senior hospital management during the first two waves of the COVID-19 pandemic in Spain. Fourteen clinical nurses who worked on the front line in inpatient units or intensive care units of the Health Services of Extremadura and Madrid in the first (March–April 2020) and second (October–November 2020) waves of the COVID-19 pandemic participated in this study. The data was collected through semi-structured interviews, following a script of themes, in a theoretical sample of nurses who were worked during the pandemic.

**Results:**

Two main themes emerged from the analysis of the data: (1) perceptions about the performance of nurse managers and senior hospital managers during the first and second waves of the pandemic (health system failure; belief that senior hospital management professionals could have managed the pandemic better; recognizing the efforts of middle management (nursing supervisors); insufficient institutional support) and (2) strategies employed by nurses to compensate for the weaknesses in pandemic management.

**Conclusions:**

The clinical nurses perceived that the nurse managers demonstrated better management of the pandemic than the hospital’s senior management, which they attribute to their proximity, empathy, accessibility, and ability to mediate between them and the senior management. The nurses also believe that the senior management of the hospitals was to blame for organisational failures and the poor management of the pandemic.

## Background

The disease caused by the new coronavirus (COVID-19), which was declared a global pandemic by the World Health Organization [1] on March 11, 2020, forced the Spanish government to publish Royal Decree 463/2020 which established a state of alarm due to the health crisis [2]. During the first weeks of this crisis, issues with the health system such as differences in the availability of resources across different regions, the lack of long-term health investment, and a failure to have previously worked on health coordination between regional and national governments became evident [3].

According to scientific evidence before the COVID-19 pandemic, nurses had already been warned that they were not prepared to face any pandemic. Administrations should provide their workers with coping strategies to mitigate the negative effects that such a potential catastrophe could cause [4].

During the first wave of the pandemic, Spanish nurses highlighted the lack of personal protective equipment (PPE), as well as poor training, planning and organization of health services, together with the strong psychological and physical impacts they suffered while caring for patients during the COVID-19 pandemic [5].

With scarce PPE, front-line nurses had to reuse what they did have several times, or use equipment that had not passed regulatory approval, increasing the likelihood of infection and their perception of risk [6]. Many nurses were recruited and reassigned to COVID-19 units, where they experienced a lack of preparation, absence of protocols and lack of safety [7], having to learn continuously [8] due to the absence of knowledge about the new disease. The combination of these aspects triggered serious psychological problems in front-line nurses during the pandemic [9, 10].

As previous studies have shown, one of the major demands of healthcare professionals during the pandemic was the improvement of working conditions and institutional support [11, 12]. Thus, according to healthcare professionals, healthcare managers should have supported their workers and organized working hours and available human resources more fairly. Nurse managers and senior hospital management ought to have engaged in effective communication with their workers in order to understand their needs and provide support. To improve this relationship, communication should have been two-way and very clear, so that both sides were aware of the challenges being faced [13]. Nurse managers (inpatient unit or ICU supervisors) are nurses closer to clinical nurses in the chain of command and perform functions of material management, shift management, and work distribution, but have no power over hiring or general organizational decisions, with the general guidelines and hiring policies, etc. being set by senior management.

The situation caused by the pandemic confronted professionals in senior hospital management positions with a variety of management challenges [14–16] such as the constant changes in the organization of services and the management of health professionals [16], the need to take quick decisions, to improvise responses to situations due to the absence of protocols, and to adapt to changes in the organization of the health professionals due to the high incidence of sick leave among them [17]. Similarly, middle management, such as nursing supervisors, faced their own challenges: having to organize and ration the scarce materials available, while also being responsible for ensuring the necessary individual protection systems for the nurses who depended directly on them [6].

Some of the recommendations identified in other studies to improve the approach by senior hospital management and nurse managers to the pandemic have been to analyse how similar situations have been handled previously, to employ continuous learning on the approach to pandemics and timely decision making, without neglecting the continuous contact with and provision of support to frontline healthcare professionals [18].

Previous studies that have analysed the experience of nurse managers in Spain during the pandemic [16] have shown a series of shortcomings and needs that have had a direct impact on the nurses in charge. In this sense, nurse managers should have prioritized the psychosocial needs of their team during the pandemic [15]. It is accepted that burnout and psychological disorders caused in front-line clinical nurses are multifactorial in origin [10]; however, without dismissing the contribution of colleagues and family members, the care provided by nurse managers is speculated to be quite significant among the mechanisms that are considered supportive [19]. Therefore, since decisions made by nurse managers and senior hospital management can have a positive impact on clinical nurses, it is important to understand the best way to support the nurses and provide them with the necessary resources to both care for themselves and provide the best care for patients [13].

## Methods

### Aim

The aim of this study was to investigate the perceptions of frontline nurses in intensive care units (ICU) and ward nurses about the performance of nurse managers and senior hospital management in relation to the support received, resource management and what could have been improved.

### Design and participants

#### Design

This was a qualitative study designed and analysed based on Giorgi’s descriptive phenomenological approach [20, 21]. This methodological approach was chosen to describe, using their own words, the perceptions that clinical nurses in inpatient and intensive care units have about the performance of nurse managers and senior hospital management and their ability to understand the nurses’ experiences of this phenomenon [21]. In addition, the recommendations of the Consolidated Criteria for Reporting Qualitative Research (COREQ) guidelines were followed [22].

#### Data collection

The data was collected from May to November 2020 through semi-structured interviews from an intentional theoretical sample of active nurses working in inpatient or intensive care units where people with COVID-19 were being cared for during the first two waves of the pandemic in four Spanish public hospitals (two in Extremadura and two in Madrid). These two autonomous communities were selected because they had a high prevalence of cases during the first two waves of the pandemic [23].

The participants were selected using the following criteria. Inclusion criteria: (1) nurses who had worked during the first two waves of the COVID-19 pandemic; (2) nurses who were working in inpatient or intensive care units in public hospitals in Madrid or Extremadura. Exclusion criteria: (1) nurses who had taken leave due to illness; (2) nurses who were caring for COVID-19 patients for less than two months (Table 1) [24]. Semi-structured interviews were chosen based on their ability to describe the phenomenon in the participants’ own words [24, 25]. A suitable time and place for the interview was agreed with each participant. The main researcher (male, FLE), who acted as the interviewer, had the help of a script of open topics that could arise during the semi-structured interviews, which was developed specifically for this study. The script of topics was refined throughout the study employing the constant comparison method (Table 2) [24, 25]. The semi-structured interviews were carried out face-to-face, except for four cases in which, due to the pandemic situation, the participants preferred an interview via video conferencing [24].

**Table 1:** Main characteristics of participants

Participant demographics (*N* = 14)		*n*
Age	< 30 years old	2
	30–39 years old	8
	40-49 years old	2
	> 50 years old	2
Gender	Male	3
	Female	11
Highest academic qualification	Registered Nurse	6
	Specialist	1
	Master	6
	PhD	1
Type of work	Temporary employment	9
	Fixed-term contract	3
	Permanent contract	2
Type of unit	Intensive Care	6
	Emergency	2
	Medical unit	6
Change of unit during COVID-19 crisis	Yes	3
	No	11
Years in Practice	0–4 years	2
	5–10 years	5
	11–15 years	3
	More than 25 years	4

**Table 2:** Interview topic script

- Performance of nurse managers and hospital senior management in handling the first and second waves of the pandemic in inpatient or intensive care units.
- Clinical nurses’ perceptions of the support received from nurse managers, senior hospital management and supervisors during the first and second waves of the pandemic.
- Clinical nurses’ perceptions of the material resources available in hospitals during the first and second waves of the pandemic.
- Aspects of the management of the pandemic that could be improved in the opinion of clinical nurses who worked on the front line in hospital units or intensive care units on.

Sample collection continued until data saturation, the point at which further expansion of the sample no longer provided new information [26, 27]. This was reached with 14 interviews. Interviews had an average duration of 66 min (range 36–116 min) and were audio recorded. Subsequently, the interviews were anonymized by assigning them a code and removing any data that could identify the participant. Finally, a literal transcription of each interview was made.

### Data analysis

The anonymized and transcribed interviews were analysed following the steps of Giorgi’s phenomenological method: (1) collecting and describing phenomenological data, (2) reading the whole description, (3) breaking descriptions into units of meaning, (4) transforming units of meaning, (5) identifying the essential structure of the phenomenon and (6) integrating features into the essential structure of the phenomenon [20, 28].

Triangulation of the analysis was carried out between researchers with the help of Atlas-ti 8.0 software.

### Trustworthiness

The trustworthiness of this study was ensured by following the criteria proposed by Guba and Lincoln [29]: dependability, transferability, credibility, and confirmability. To guarantee credibility and minimize the influence of researchers’ personal interpretations, we used triangulation of the analysis between researchers. Moreover, interview transcripts were returned to participants to confirm their agreement with them. In terms of confirmability, we have prioritized transparency throughout our research process. We engaged in discussion sessions to establish consensus on codes, categories, and emerging themes. To ensure study dependability, we provided detailed information about the research design, implementation, data collection, and analysis. In addition, we have meticulously described the participants’ characteristics and the research environment [24].

## Results

A total of 14 clinical nurses (11 women and 3 men) with a range of academic qualifications, years in practice, types of work, and ages, all of whom worked on the front line in inpatient units or intensive care units of the Health Services of Extremadura and Madrid in the first (March–April 2020) and second waves of the COVID-19 pandemic participated in this study. Six of the nurses faced both waves and eight faced the first wave. Table 1 outlines the main characteristics of the participants. Nine of the participants were from hospitals in Extremadura and five were from Madrid. No differences were found in the nurses’ perceptions of the study phenomenon according to place of work, years of experience, gender, or the autonomous community where they worked. Moreover, no differences were found in the themes that emerged in face-to-face and video conference interviews.

Two main themes emerged that explained the perceptions of clinical nurses: (1) perceptions of the performance of nurse managers and senior hospital management and (2) strategies to address the weaknesses in the management of the pandemic. Within each theme, the main categories, subcategories and codes were identified; these will be explained below and are summarized in Tables 3 and 4. To facilitate the understanding of the results, the codes are accompanied by a selection of the main verbalisations of the participants in Tables 3 and 4.

**Table 3:** Theme 1: perceptions of the performance of senior hospital management and nurse managers during the pandemic

Categories	Subcategories	Codes	Verbatims
Health System Failure		Lack of resources, materials, and personnel protection of nurses	"Every death is not a failure on our [nurses’] or the doctors’ part, it’s just that we couldn’t do anything else. Some patients needed to be transferred to the ICU for ventilation and you knew they were going to die if they weren’t. It is a failure of the health system. It’s bad luck because if it had happened a fortnight ago there would have been ventilators and ICU beds for them, and expert professionals on hand, and now there aren’t..." (P. 10M30).
			"They didn’t even know how to keep us safe; the protective material was scarce and of very poor quality..." (P.01H24).
The belief that senior hospital management professionals could have managed the pandemic better	Areas where the management of the pandemic was inadequate	Lack of foresight	"They [senior hospital management] have shown that they have not been effective. It has been very hard for all of us, but they have lacked a great deal of foresight and could not see what was coming. And apart from that, the tremendous calmness, the idleness, of doing absolutely nothing, of standing by and watching made me very anxious. When other people were already sounding the alarm and nothing was being done, no measures were being taken, and there was nothing (neither material nor personnel)... (P. 07M32)
		Organisational chaos	“In the unit, there was a lot of lack of coordination due to misinformation – we were like chickens with their heads cut off. One day they would tell you one thing and the next they would tell you something else. One day the care protocol would say one thing, and the next day it would have changed. One day we would put those who might be infected in individual rooms, the next we would put them all together. Then we started to organize ourselves and to improve. For example, we started to ask the pharmacy to prepare sedations in large quantities so that perfusions would last for a day” (P. 05H30).
		Poor information management	"The government telling us one thing and the next day something different... It has been like a battle. They said one thing and you say “how can that be?”, it was the opposite of what those in charge were saying. It seemed like we were being led by monkeys. Also, the source of the information, you say “how can it be like that, have you thought about this before saying it?” "They told us that the surgical mask gave the same protection as FFP2 ... you say “you don’t need to lie”. We’re not that stupid either, you know? Say that there isn’t any, that’s all there is, because I don’t think we’re going to walk out without taking care of the patients, we’re going to help them anyway, but just be honest and that’s it" (P. 05H30).
	Management of health professionals	Lack of foresight in the number of professionals	"At the beginning, there was a lack of “hands on deck”. We have a very low ratio in this ICU, and we asked for more staff because we knew what was coming, and the staff sent to us was scarce and had barely any ICU experience... There was a lack of foresight" (P.07M32).
		Unit switches	"We were moved to another unit, and we had no training in how to put on PPE, nor how to work in ICU, or the use of respirators. At that time nothing was known about how the disease evolved, how it affected us..." (P. 08H32).
		Changes in quarantine criteria for nurses	"Nurses are indeed necessary, they are needed because if we all went into quarantine there would be no one to look after the patients, but what you can’t do is say one thing and when there is a surplus of staff say “No, no, you stay at home. Stay in quarantine, don’t worry” and when they needed people... they would tell you ... “come on, nothing will happen”. I don’t like that” (P. 05H30).
	Material resources	Lack of material	"We had no protective equipment at the start. The main thing we had was, as you may have seen in pictures on Facebook and social media, a lot of rubbish bags. We didn’t have screens, ... We bought our goggles at the hardware store" (P. 03M38).
		Material without regulatory approval	"... they gave us masks that were not approved, which had to be withdrawn. So they withdrew them, but as quickly as the management could, it took about 3 days to delay the material and you continued to use them because there was nothing else... Then you asked yourself “is what I’m wearing any good?”. You look at the patient and you say... “really... I'm not passing something on or the patient or the patient is passing it on to me because the material is not good?” (P.02M45).
		Re-use of material	"We didn’t have enough material. We have been using the masks for ten days. The good PPE didn’t come until mid-April or so. What did come were some gowns from the Chinese bazaar that we would reuse. When we left the rooms, we would spray the gowns with a mixture of bleach, and we would hang them out and use them again" (P.06M52).
	Training resources	Self-training on social networks, internet or with other partners	"We did not have any training... We searched the internet (social networks, YouTube, etc.) to find out how to manage these patients, what they needed, to look for experiences of colleagues from other hospitals, how they were coping... nobody (senior management) trained us in anything, neither in how to receive patients, nor protocols on anything... it was like a “come on, get on with it. You can do it” " (P.07M32).
		Training outside working hours	"My days off were spent watching videos on the internet about ventilators, watching a thousand protocols, and training myself daily. I had to leave the ICU and look for information because since I was isolated, I didn’t switch off for 24 hours thinking about “what can I do to improve this?” (P.07M32).
	Care and intervention protocols	Own protocols	"We started very disorganised due to a lack of information; at the beginning we had no protocols, and we began to prepare them, to make videos. We started to organize ourselves to try to enter the area with the patients as little as possible, to stay as little as possible, and I think that after about twenty days we were well organized. We found tricks to avoid fogging up our goggles, to take breaks, to organize the perfusions, we wrote down on a list that we stuck on the windows how much time was left for each perfusion so that no colleague would be too exposed and that at a glance they would know whether or not they had to go in to change it, we left everything prepared between some colleagues and others..." (P. 07M32).
Recognition of the efforts of middle management (nursing supervisors)		Good management	"Our supervisor was always on top of everything, making sure there was no shortage of material, keeping us up to date, the staff, the staffing tables, etc. Even when upper management told us not to use masks or PPE, because they still didn’t know what it was... but thanks to our supervisor we always had respiratory isolation material in the unit. He made a special WhatsApp group for COVID-19, where the supervisor passed on all the information and communication was more immediate and continuous. ... despite his complex position between us and those above us, I take my hat off to him for his performance... without him, things would have been worse..." (P. 02M45).
		Nurse Supervisors as a link to the team of professionals	"The nursing supervisor was like the bridge, she kept the ward together, even though there were a lot of new people in the ward she kept us together as a team even though we had not worked together..." (P.02M45).
		Closeness, readiness, empathy, and visibility	“Our supervisor was there for us in whatever we needed, she cared about us emotionally, she was there to cry together, to say a kind word to you, if you looked bad and she could give you days off, she did... she showed empathy and was always in the unit... she was there, with us at the bottom of the barrel..." (P.09M52).
Insufficient institutional support		Failure to recognize their work and the risk to their well-being.	"The helplessness, the anger of saying “they don’t think about us”. What we do for people is not being appreciated, society doesn’t value it, nor do the administrations. In addition, nurses and auxiliary staff were not tested for antigens until we had symptoms... On the other hand, doctors were tested without any symptoms or anything. You feel like a pawn... it seems that your life isn’t worth as much or that it matters less... we have been ignored by the authorities..." (P. 05H30).
		Their well-being and safety were worth less than that of the physicians.	"... some lives were worth more than others. Because when there was no FFP2 for us, for the few moments the doctors came in, which might be ten minutes, they came in with FFP2. They were given better material, with more comfortable goggles for the five minutes they came in, and we had ulcers and marks on our faces because we had goggles that were the same for everyone. It was our material and that was it. For me, those details hurt me a lot because I didn’t expect that discrimination" (P. 07M32).
		Deception	"To tell the truth, I don’t know if I would work with COVID-19 in a second wave. I see that what we do for people is not valued, neither the people nor the administration value it. Everything has been forgotten. The nurses save their lives and they have forgotten everything in a second. I love my profession, but I am disappointed that the institutions do not value it in that way” (P. 05H30).

**Table 4:** Theme 2: strategies to respond to weaknesses in the management of the pandemic

Categories	Codes	Verbatims
Developing their own protocols of care and action	Developing their protocols at the onset of the pandemic	“We started out very disorganized due to the lack of information, we began to prepare protocols practically by leap and bounds, we began to make videos, to do things about protocols to try to enter as little as possible with the patients, to spend as little time as possible right next to them, …” (P. 07M32).
Self-training	Self-training outside working hours	“The Administration has not provided any training as such through courses. It has all been either a personal effort of watching videos and reading at home; or with work colleagues who accompanied you in putting on and taking off PPE and gave you instructions…” (P. 01H24).
Demanding equality in the distribution of equipment	Inequity in the distribution of materials	“…we arrived and there was no FFP2 and we said “we’re not going in until you bring them up” and there were some nurses who were gave more of a fight who said “I won’t go in until you get me PPE” because they knew there was some on other floors and then they were like “if there is some on other floors, it’s good enough for me too. If there isn’t one for anyone else, fine, but if they have it in other places, there should be for me too” " (P. 10M30).
Purchasing their own protective equipment	Self-supply of protective material	“At the beginning, we were not given the gowns and goggles, but because the pandemic had not yet been officially declared… Many people bought anti-splash goggles and there are people who even when you see them now have their own goggles that they bought and they disinfect them themselves because of the uncertainty of being in a place where there may not be material… this floor has finally got material not only because of management, but because we have brought a lot of material into the floor ourselves” (P. 02M45).

### Theme 1: perceptions of the performance of senior hospital management and nurse managers during the first two waves of the pandemic (Table 3)

The perceptions of clinical nurses who worked on the front line in inpatient or intensive care units were grouped into the following categories: failure of the healthcare system, belief that senior hospital management professionals could have managed the pandemic better, recognition of the efforts of middle management (nursing supervisors), feeling abandoned by superiors, and feeling like pawns of the healthcare administration. Some of the categories included subcategories. Each category included a series of codes that are described in each section and Table 3.

#### Health system failure

Nurses felt that the health system had failed to manage the pandemic because of a lack of resources to protect them and treat patients fairly. This was attributed to a shortage of health professionals, lack of equipment or resources such as ventilators and ICU beds to care for patients, lack of personal protective equipment and shortage of nurses.

#### The belief that senior hospital management professionals could have managed the pandemic better

Despite the initial lack of knowledge and the evolution of the virus, nurses felt that senior hospital management professionals could have managed the onset of the pandemic better.

Nurses highlighted the following subcategories where the management of the pandemic was inadequate:

##### Lack of foresight

Senior hospital management professionals should have foreseen how the pandemic would evolve, as other countries in Europe (e.g. Italy) were already experiencing a devastating situation. Because of this, the nurses felt that protocols and contingency plans could have been drawn up, staffing levels could have been increased, and protective materials could have been procured well in advance. In addition, they perceived that on many occasions senior hospital management acted too calmly or even carelessly at the onset of the pandemic.

##### Organisational chaos

Nurses highlighted the lack of coordination and the initial disorganisation of work in the inpatient units, which left them with a sense of chaos at the beginning of the pandemic.

##### Poor information management

Nurses felt that senior hospital management professionals did not handle information correctly at the beginning of the pandemic, withholding information from frontline health professionals or providing it to them at the wrong time.

In addition, nurses perceived that the information they received from hospital management at the beginning of the pandemic was erratic, untruthful, and unreliable, which generated mistrust and unease among nurses, as they felt they were being treated as if they were children or lacked intelligence. Nurses felt that they would have been more accepting if senior hospital management had told them that there was no material and that they did not know how the pandemic would evolve.

##### Management of health professionals

The nurses felt that health professionals could have been managed better in several respects. Firstly, they felt that there was a lack of foresight in the number of professionals that would be needed. Secondly, they were liable to be reassigned overnight to a new work unit, or expected to start new contracts without any training. Thirdly, even key aspects such as whether to quarantine after contact with a positive case depended on the demand for health professionals in each hospital.

##### Material resources

At the beginning of the pandemic, the lack of protective material and having to reuse the little protective material available was one of the aspects most frequently mentioned by the nurses as needing to be improved. In addition, the nurses stressed that at certain times they were issued with material that had not been subject to regulatory approval and had to reuse items of personal protective equipment, putting their physical well-being and safety at risk.

##### Training resources

According to the nurses’ responses, the feeling of a lack of institutional support extended to such basic issues as training. In this regard, the nurses highlighted the lack of formal training provided by the hospitals at the beginning of the pandemic, which led them to self-training. Among the sources of information used, the clinical nurses highlighted social networks such as Twitter, YouTube, WhatsApp groups, and other internet resources. In addition, they turned to colleagues from other hospitals. As for the timing of the self-training, the nurses emphasized that it was carried out during their free time or outside their working day.

##### Care and intervention protocols

At the beginning of the pandemic, in the absence of protocols for action defined by hospital management, it was the nurses who created their own protocols for handling the situation and resolving the problems that arose, which they subsequently adapted to care needs as the pandemic evolved.

#### Recognition of the efforts of middle management (nursing supervisors)

Most of the nurses praised the management by their supervisors at the beginning of the pandemic, acknowledging the hard job they had to do; receiving changing orders from hospital management, the frustration of not being able to provide the professionals in their unit with protective material or having to manage the scarce material that arrived, managing the professionals in their units, who were often new, had not worked together or had little experience, and training the professionals in their unit with the little they knew.

The early days of the pandemic were characterized by chaos and a lack of information and materials. The clinical nurses reported that some conflicts arose with their supervisors because they had different perspectives of the situation they were experiencing. According to the clinical nurses, the supervisors defended the vision conveyed to them by the hospital’s senior management, which differed from the experience of the nurses who were facing COVID-19 on the front line. Thus, one of the main conflicts arose over the use of masks in the first days of the pandemic. Despite the above, the nurses felt that the supervisors had to do what senior management instructed them to do in the chaotic first days. The nurses also felt that, due to their involvement, empathy, availability, and presence as a visible authority during the pandemic, they were closer to the health professionals than the hospital senior management. This closeness, empathy and availability was associated with the perception by the clinical nurses that the supervisors had managed the pandemic better than the senior hospital management.

The clinical nurses also highlighted the work of their supervisors as liaisons between professionals in the different units, as well as their dedication, commitment to the team of professionals, and the emotional support they provided.

#### Insufficient institutional support

During the first and second waves of the pandemic, nurses perceived that although they were risking their well-being, on many occasions they did not receive the institutional support from senior hospital management that they would have liked. In addition, the clinical nurses highlighted the lack of appreciation and recognition of their work by the health administration, to the extent that they felt as if they were being treated as labourers or expendable. The nurses also had the perception that the lack of guidelines from senior hospital management, the lack of training provided by the hospitals or health administration, the rapid and changing information transmitted, and the lack of material made them feel abandoned by their superiors and without support, being left to look for information and organize themselves to handle the situation. In addition, they felt that on many occasions the health administration did not value their physical well-being and safety, due to the practice of modifying quarantines according to the number of health professionals available to the hospitals during the pandemic, by limiting access to diagnostic tests to detect the virus, and by providing nurses with inferior protective equipment than that given to doctors, even though the nurses were the first line of care for patients 24 h a day.

Furthermore, the clinical nurses highlighted that the health administration also failed to recognize, both in financial terms and in terms of improved working conditions, the risks to which they were exposed during the pandemic.

According to the nurses’ responses, their disappointment with the health administration led them to consider not working in subsequent waves of the pandemic.

### Theme 2: strategies to respond to weaknesses in the management of the pandemic (Table 4)

Within this theme, we identified the following categories that explain the strategies implemented by nurses in response to the weaknesses they identified in management during the pandemic: developing their own protocols for care and action; self-training; demanding equality in the distribution of equipment; and acquiring protective equipment themselves. Each category includes a series of codes that are described in each section and in Table 4.

#### Developing their own protocols of care and action

Disorganization, initial chaos, and the absence of written protocols from hospital management led clinical nurses to create their own protocols for action and care to organize their work in the early stages of the pandemic.

#### Self-training

Facing a new and unfamiliar working situation, coupled with the lack of formal training from health authorities at the beginning of the pandemic, nurses were forced to spend much of their free time on self-training.

#### Demanding equality in the distribution of equipment

Given the inequity in the distribution of protective equipment among different units and services of the hospital and even among different professional categories, the nurses demanded that the equipment be distributed equally.

#### Purchasing their own protective equipment

To cover the initial shortage of protective equipment, the nurses went so far as to buy their own protective equipment, especially goggles and splash shields. Although this material was bought from hardware stores and was therefore had not passed regulatory approval, they felt it was better than nothing at all.

## Discussion

This study analysed clinical nurses’ perceptions of the performance of healthcare managers during the first and second waves of COVID-19, with two main themes emerging: (1) perceptions of the performance of nurse managers and senior hospital management, and (2) the main strategies deployed by nurses to address the shortcomings in management of the pandemic during the first two waves.

In the opinions of the nurses, healthcare organizations and senior hospital management failed to provide safe working conditions, sufficient material resources, or adequate rewards to clinical nurses who have cared for people with COVID-19 [30]. Our results are consistent with another study which noted that clinical nurses perceive that hospital senior management did not adequately manage the pandemic. Moreover, as previously noted, the nurses saw the pandemic situation as an unprecedented phenomenon in which the work atmosphere was constantly changing, which necessitated conscientiousness and flexibility from the nurses [31, 32]. One of the new findings from our study is the positive appraisal of middle management’s approach to the pandemic, and that the nurses associate the involvement, empathy, and willingness of nursing supervisors with a perception of having managed the pandemic better. In contrast, the nurses perceive that the top management of the hospitals did not provide good management through the pandemic, having been distanced from the problems nurses were facing on a daily basis.

Similar to previous studies, it was reported that the context of uncertainty and organizational chaos due to frequent changes defeated any attempt at planning [14, 33]. This situation led to a constant need to design new protocols and continuously reorganize services according to new, changing and even contradictory information on COVID-19. These factors increased fear and mistrust, and, according to the perceptions of the nurses, overburdened clinical nurses and nurse managers [13, 16].

In agreement with another study, nurses felt that the circumstances of the pandemic represented a barrier for nurse managers and senior hospital management to effectively manage information and communicate between the different levels of the hospital, acknowledging that decisions had to be made quickly and sometimes with little consensus [16]. Furthermore, our results are consistent with another study which showed that supervisors and the entire nursing team were in continuous communication via instant messaging applications to keep up to date [15]. This increased the perception of the supervisors’ closeness to the situation.

Although material resources (respirators, ICU beds, protective equipment) were limited everywhere, the nurses in this study consider that there was a lack of foresight in managing the pandemic; Spain was not the first country to be affected, as the pandemic had already struck other European countries such as Italy a few weeks earlier, foreshadowing what could happen in Spain [12, 34, 35].

As in previous studies, nurses associated the pandemic situation with an increase in the intensity and volume of their work, leading to increased burnout and inefficient management [8, 24]. Nurse managers had to cope and manage with a shortage of staff, or without adequate training to handle the pandemic [14, 16].

Our results are consistent with other studies that have shown that nurses engaged in constant training to provide more efficient and safer care, without losing sight of the fact that the patient was at the centre of care [35, 36]. In addition, our study highlights the predominance of self-training by clinical nurses in Spain, as hospitals and health organizations did not provide any training in the initial stages of the pandemic. The nurses attributed this to a lack of foresight.

The whole pandemic situation described above sometimes led to conflicts between nurse managers, senior hospital management, and clinical nurses. This, in turn, led to the clinical nurses blaming the former two groups for the chaos and ineffective management, as also found by White [15]. Thus, our results are in agreement with previous studies [37, 38], which state that hospital managers either lacked the knowledge on how to manage the crisis or the experience to handle the situation. This reinforces the idea that senior managers need better training in disaster management, and more organizational support [16, 37, 39].

Despite this, the nurses we interviewed also appreciated the effort and work of the majority of middle managers and nursing supervisors, highlighting that they were available, approachable, closely involved, and empathetic to the challenges of providing care in times of pandemic. Some studies have highlighted how the work of nurse managers may have led to different experiences of the pandemic, depending on their personal involvement and leadership skills [38, 40, 41]. As noted previously, many nurse managers were conscious of the impact of the COVID-19 pandemic on the healthcare professionals in their charge and supported them emotionally, having a significant effect on reducing the anxiety and improving the mental well-being of the professionals under their care. These factors also contribute to the increased confidence of healthcare professionals in their managers [14–16, 31, 38, 40, 41].

Of note in our study is the perception of a lack of support or protection from the healthcare organization; a fact that may have increased the psychosocial impact of the pandemic on clinical nurses (their fear, psychological distress, dissatisfaction with work and thoughts of leaving the profession), as also reported by other studies with similar results [10, 12, 32, 42, 43]. Therefore, our results agree with the findings of Danielis [7], that determined and strong support for frontline nurses should be a priority for senior management professionals and nurse managers. Moreover, the nurse managers themselves would also like more support [14].

Another important finding is the impression held by clinical nurses that physicians received more support and protection from senior hospital management and the healthcare administration, which other studies have characterized as a lack of respect for nurses [31, 38, 44].

Our results show that self-education has been constant throughout the pandemic, being perceived as a very good coping strategy, and nurses were more satisfied the more experienced and knowledgeable they became about the virus and how to deal with it [32].

In another previous study, nurses reported sometimes having to purchase their own equipment or being unsupportive of colleagues in other units by not lending them protective equipment [45].

Finally, our findings agree with those of another study [46] that highlighted the importance of having good managers who employ appropriate leadership strategies, both during and after the pandemic, to improve the psychological and emotional well-being of nurses.

### Strengths and limitations

Our research assessed the perceptions of clinical nurses in ICU and inpatient units; therefore, it is not possible to generalize these findings to other contexts. Furthermore, this study only addressed the perceptions of nurses during the first wave or the first two waves of the pandemic. Future studies should analyse whether nurses’ perceptions changed in different waves of the pandemic and in the long term. The fact that some nurses had to pay for their EPI resources makes it necessary for future studies to analyse whether the socioeconomic level of nurses could have influenced their working and health conditions.

One of the strengths of this study is that it analysed nurses’ perceptions of the first waves of the pandemic, which were marked by uncertainty and lack of knowledge, and necessitated innovative approaches to resolving management conflicts.

### Relevance to clinical practice

Closeness, empathy, and engagement of nurse managers are associated with a perception of better management of the pandemic. Senior management of the hospitals has been blamed for organizational failures and poor management of the pandemic. Determined and strong support for frontline nurses should be a priority for senior management professionals and nurse managers. Nurse managers must be in a position to support the clinical nurses psychologically and organizationally to ensure the latter can perform their work safely and effectively.

## Conclusions

Clinical nurses believe nurse managers demonstrated better management of the pandemic, being available and involved and showing empathy for the situations the nurses were experiencing.

The clinical nurses clearly differentiate between the work of nurse managers (closer to them) and senior management (more distant and in their “ivory towers”). They believe that nurse managers have been good mediators between them and the senior management of the hospital, and blame the latter for the organizational failure and mismanagement. The senior management was responsible for the guidelines and decisions that defined their working environment and work organization, sometimes inconsistently.

The COVID-19 pandemic is an example of an extreme situation where it is critical to have good and courageous leaders and nurse managers who provide the necessary psychological and organizational support that clinical nurses need to perform their tasks safely and effectively.

Nurse managers must be given the tools to better manage future pandemics.

## Data Availability

The data that support the findings of this study are available from the corresponding author upon reasonable request.

## Abbreviations

COREQ: Consolidated Criteria for Reporting Qualitative Research
ICU: Intensive Care Unit(s)
PPE: Personal Protective Equipment
